# Sunitinib-Induced Acute Interstitial Nephritis in a Thrombocytopenic Renal Cell Cancer Patient

**DOI:** 10.1155/2017/6328204

**Published:** 2017-11-22

**Authors:** Ibrahim Azar, Saghi Esfandiarifard, Pedram Sinai, Ali Wazir, Llewellyn Foulke, Syed Mehdi

**Affiliations:** ^1^Department of Internal Medicine, Albany Medical Center, 47 New Scotland Ave., Albany, NY 12208, USA; ^2^Department of Pathology, Albany Medical Center, 47 New Scotland Ave., Albany, NY 12208, USA; ^3^Division of Hematology and Oncology, Department of Internal Medicine, Stratton Veterans Affairs Medical Center, 113 Holland Ave., Albany, NY 12208, USA

## Abstract

Sunitinib, a multitargeted tyrosine kinase inhibitor (TKI), is currently the standard of care for patients with metastatic renal cell carcinoma. Renal adverse events associated with sunitinib include proteinuria, renal insufficiency secondary to focal segmental glomerulosclerosis (FSGS), and thrombotic microangiopathy. We describe the second reported instance of biopsy-proven sunitinib-induced acute interstitial nephritis (AIN), in a challenging case complicated by thrombocytopenia. The case illustrates the importance of early diagnosis and intervention in ensuring long-term recovery from renal complications. Four other cases of AIN reported along with inhibition of the vascular endothelial growth factor (VEGF) by either TKI (sunitinib and sorafenib) or antibodies (bevacizumab) suggest a possible class effect. Given our experience, we recommend monitoring renal function with VEGF inhibition, and in the case of renal failure in the setting of an unclear diagnosis, we recommend prompt biopsy.

## 1. Introduction

Recently, the use of tyrosine kinase inhibitors (TKIs) as antineoplastic agents has increased exponentially. The current National Comprehensive Cancer Network (NCCN) [1] and the European Association of Urology (EAU) [2] guidelines recommend the use of the vascular endothelial growth factor (VEGF) TKI sunitinib [2], among other options (pazopanib, interferon, and bevacizumab), as first-line treatment for metastatic renal cell carcinoma (mRCC). Given their oral availability and their generally well-tolerated side-effects profile, these antiangiogenesis agents have become the standard of care. Despite their widespread use, our understanding of this relatively new class of drugs and their side-effect profile is rudimentary.

Acute interstitial nephritis (AIN) is a characteristic inflammatory infiltrate in the renal tubule-interstitium that is a leading cause of acute renal failure. Drug-induced injuries are thought to be responsible for 60–70% of cases [3], with systemic, autoimmune, and infectious diseases accounting for the rest. In this report, we describe a case of biopsy-proven AIN in an mRCC patient receiving sunitinib.

## 2. Case Report

A 69-year-old male with a history of stage IV left kidney clear cell carcinoma with metastases to spine and brain presented with a four-day history of gross hematuria, oliguria, fever of 38.5°C, fatigue, and a decreased appetite. He had been started on sunitinib (50 mg daily at bedtime) two weeks ago. Hematuria persisted despite stopping acetylsalicylic acid/dipyridamole prescribed for a previous stroke (for over 5 years), discontinuing sunitinib and a course of amoxicillin for presumed UTI. Upon presentation to ED, vitals were significant for a blood pressure of 160/72 mmHg. A digital rectal exam was negative for occult blood. Laboratory investigations revealed mild anemia (hemoglobin of 11.9 g/dl), thrombocytopenia (platelets of 68,000/mm^3^), hyponatremia (sodium of 120 mEq/L), and acute renal failure (BUN of 41 and creatinine of 2.7 mg/dL with a baseline creatinine of 1.0). Coagulation labs were within normal limits (activated partial thromboplastin time (aPTT) 29, prothrombin (PT) 12.1, and international normalized ratio (INR) 1.1). A urine dipstick was positive for blood and protein. Urine sodium was less than 10 mEq/L, indicating dehydration. Total urine protein collected in 24 hours was 484 mg. Haptoglobin was not decreased, and a peripheral smear examination was negative for schistocytes, ruling out hemolytic uremic syndrome. While nonspecific inflammatory markers (C-reactive protein (CRP) of 55 mg/L and erythrocyte sedimentation rate (ESR) of 80 mm/hr) were elevated, multiple serologies associated with nephritic syndromes (peripheral and cytoplasmic antineutrophilic cytoplasmic antibodies (p- and c-ANCA): antimyeloperoxidase (MPO), antiproteinase 3 (PR3), complement levels, antinuclear antibody (ANA), anti-double-stranded DNA (dsDNA), rheumatoid factor (RF), hepatitis B and C panels, and cryoglobulins) were all negative.

**Figure 1:**
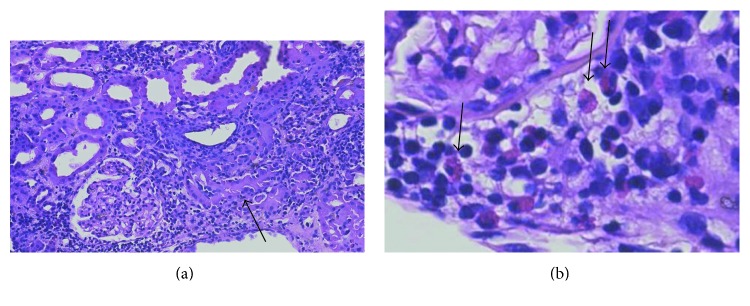
Renal biopsy demonstrating acute interstitial nephritis (H&E staining): there is edema and an extensive dense interstitial chronic inflammatory infiltrate in the cortex and medulla. (a) In the lower power photomicrograph (left, 100x), the renal cortex shows extensive interstitial chronic inflammation with occasional lymphocytic tubulitis (arrow). In areas of inflammation, there is evidence of acute tubular injury with proximal tubules demonstrating lumenal distension with epithelial flattening. There was no evidence of glomerulitis, vasculitis, or thrombotic microangiopathy. (b) The higher magnification photomicrograph (right, 600x) shows that the interstitial inflammatory cells comprised frequent eosinophils (arrows) as well as lymphocytes and fewer plasma cells.

**Figure 2:**
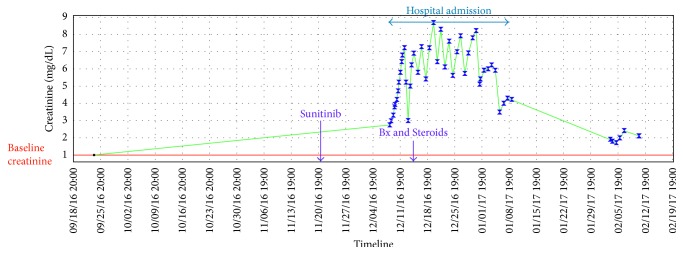
Timeline showing the temporal relationship between the start of sunitinib and the rise of creatinine. Note the normal creatinine at baseline and the decrease of creatinine with steroids and hemodialysis.

Despite a significant risk of bleeding, a renal biopsy after platelet transfusion was performed to aid in diagnosis. The renal biopsy (Figure 1) showed extensive interstitial inflammation with frequent eosinophils and interstitial edema, consistent with drug-induced acute interstitial nephritis (AIN). The inflammation was more marked in the cortex than in the medulla. There was associated acute tubular injury in regions of inflammation. There were occasional foci of lymphocytic and eosinophilic tubulitis. There was minimal chronicity evident. No evidence of glomerulitis or vasculitis was present. No evidence of immune complex deposits was identified on direct immunofluorescence studies or on ultrastructural analysis. No evidence of thrombotic microangiopathy was present. He was started on oral course of steroids and required intermittent hemodialysis. Throughout his hospital stay, he continued to experience gross hematuria requiring multiple pRBC transfusions. Unfortunately, while the patient's renal function somewhat recovered (Figure 2), he succumbed to hospital-acquired pneumonia 2 months after his diagnosis of AIN.

## 3. Discussion

This report describes an exceptional diagnostic challenge. The initial presentation of hematuria, thrombocytopenia, and renal failure raised the flag for a thrombotic microangiopathy (TMA), namely, hemolytic-uremic syndrome (HUS), a well-described side effect [4] of VEGF inhibition. However, the absence of schistocytes on the peripheral smear along with the normal haptoglobin pointed away from this diagnosis. Similarly, the constellation of hematuria and mild proteinuria pointed to a possible nephritic disease presentation. However, multiple serologies of systemic diseases associated with nephritic pattern pathology were negative. In the setting of a worsening renal failure requiring dialysis and an uncertain diagnosis, the decision to obtain a kidney biopsy was contemplated. However, the unabating thrombocytopenia exacerbated the risk of retroperitoneal hematoma, the most common side effect of kidney biopsy, to 40% according to one study [5]. After extended discussions between oncology, nephrology, and interventional radiology, the kidney biopsy was performed and revealed the diagnosis of AIN.

Interestingly, when faced with a patient with thrombocytopenia and renal failure following the administration of sunitinib, Khurana [6] decided against performing a biopsy. A notable diagnostic difference between the two cases is that of the classically described AIN triad (rash, fever, and eosinophilia); our case presented with the very nonspecific fever, while the patient seen by Khurana had a peripheral eosinophilia of 40%. The presumed diagnosis of AIN in the Khurana case was further strengthened when renal failure eosinophilia recurred when the patient was rechallenged with sorafenib, another VEGF inhibitor. Unfortunately, despite recovering some kidney function with the course of corticosteroids, our patient succumbed to pneumonia briefly afterwards. Given our experience, we recommend monitoring renal function with VEGF inhibition and advocating a prompter biopsy and treatment in cases of renal failure.

Given the patient use of acetylsalicylic acid/dipyridamole for over five years, interstitial nephritis secondary to acetylsalicylic acid and dipyridamole was on the differential. However, the corresponding biopsy would show evidence of chronic interstitial nephritis, that is, increased interstitial fibrosis and tubular atrophy that is more than what is expected for the patient's age. However, the biopsy demonstrated minimal interstitial fibrosis and tubular atrophy (estimated to be 5–10%) less than the 25–35% expected with a 69-year-old gentleman. As such, acetylsalicylic acid/dipyridamole as the offending drug was ruled out.

This report describes the second known case of biopsy-proven sunitinib-induced acute interstitial nephritis [7]. Current techniques in rational drug design do not allow for the extreme specificity in the era of personalized medicine promised. While sunitinib was ostensibly designed as a VEGF inhibitor, it is in fact a multikinase inhibitor that includes the following receptor tyrosine kinases (RTKs) [8, 9] among its targets: platelet-derived growth factor receptor (PDGFR), stem cell factor receptor c-kit, FMS-like tyrosine kinase 3 (FLT3), and colony-stimulating factor 1 (CSF-1). Similar case reports involving the VEGF-targeting drugs sorafenib (TKI) [6, 10] and bevacizumab (monoclonal antibody) [11, 12] imply a class effect specific to VEGF. No similar case reports were found with other targets. The incidence of AIN with nonrenal cancer [11, 12] indicates that the presence of underlying renal oncogenesis is not associated with AIN.

Previously reported renal adverse events of sunitinib include hypertension, proteinuria, renal insufficiency, and thrombotic microangiopathy; all are present in this case except thrombotic microangiopathy. Thus, thrombocytopenia in this case may be secondary to bone marrow suppression or immunologic in nature [13].

While sunitinib-induced AIN is exceedingly rare, it should be considered in patients with acute renal failure. Given the multiple case reports implicating VEGF-targeting drugs in cases of AIN, further study is required to elicit the link between the VEGF pathway and AIN.

## References

[1] National Comprehensive Cancer Network Kidney cancer (version 2). https://www.nccn.org/professionals/physician_gls/PDF/kidney.pdf.

[2] Ljungberg B., Bensalah K., Canfield S. (2015). EAU guidelines on renal cell carcinoma: 2014 update. *European Urology*.

[3] Perazella M. A., Markowitz G. S. (2010). Drug-induced acute interstitial nephritis. *Nature Reviews Nephrology*.

[4] Bollée G., Patey N., Cazajous G. (2009). Thrombotic microangiopathy secondary to VEGF pathway inhibition by sunitinib. *Nephrology Dialysis Transplantation*.

[5] Simard-Meilleur M. C., Troyanov S., Roy L., Dalaire E., Brachemi S. (2014). Risk factors and timing of native kidney biopsy complications. *Nephron Extra*.

[6] Khurana A. (2007). Allergic interstitial nephritis possibly related to sunitinib use. *American Journal of Geriatric Pharmacotherapy*.

[7] Winn S. K., Ellis S., Savage P., Sampson S., Marsh J. E. (2009). Biopsy-proven acute interstitial nephritis associated with the tyrosine kinase inhibitor sunitinib: a class effect?. *Nephrology Dialysis Transplantation*.

[8] Chow L. Q. M., Eckhardt S. G. (2007). Sunitinib: from rational design to clinical efficacy. *Journal of Clinical Oncology*.

[9] Papaetis G. S., Syrigos K. N. (2009). Sunitinib: a multitargeted receptor tyrosine kinase inhibitor in the era of molecular cancer therapies. *BioDrugs*.

[10] Izzedine H., Brocheriou I., Rixe O., Deray G. (2007). Interstitial nephritis in a patient taking sorafenib. *Nephrology Dialysis Transplantation*.

[11] Barakat R. K., Singh N., Lal R., Verani R. R., Finkel K. W., Foringer J. R. (2007). Interstitial nephritis secondary to bevacizumab treatment in metastatic leiomyosarcoma. *Annals of Pharmacotherapy*.

[12] Lomax A. J., Hill P. A., Ashley D. M. (2013). Case report of interstitial nephritis induced by bevacizumab therapy for glioblastoma multiforme. *Journal of Oncology Pharmacy Practice*.

[13] Shekarriz R., Koulaeinejad N., Nosrati A., Salehifa E. (2015). Sunitinib induced immune thrombocytopenia. *Iranian Journal of Pharmaceutical Research*.

